# A phase III, randomized, non-inferiority study comparing the efficacy and safety of biosimilar filgrastim *versus* originator filgrastim for chemotherapy-induced neutropenia in breast cancer patients

**DOI:** 10.6061/clinics/2016(10)06

**Published:** 2016-10

**Authors:** Roberto Hegg, André Mattar, João Nunes de Matos, José Luiz Pedrini, Sabina Bandeira Aleixo, Roberto Odebrecht Rocha, Renato Peixoto Cramer, Sylvie van-Eyll-Rocha

**Affiliations:** I Hospital Pérola Byington, Centro de Referência da Saúde da Mulher, São Paulo/SP, Brazil; II Hospital Universitário de Brasília, Brasília/DF, Brazil; III Hospital Nossa Senhora da Conceição, Porto Alegre/RS, Brazil; IV Hospital Evangélico de Cachoeiro de Itapemirim, Cachoeiro de Itapemirim/ES, Brazil; V Hospital Santa Marcelina, São Paulo/SP, Brazil; VI Hospital Bruno Born, Lajeado/RS, Brazil; VII Instituto do Câncer Arnaldo Vieira de Carvalho, São Paulo/SP, Brazil

**Keywords:** Neutropenia, Filgrastim, Neutrophils, Non-Inferiority, Biosimilar Drugs, Fiprima®

## Abstract

**OBJECTIVES:**

To compare the efficacy and safety of two filgrastim formulations for controlling chemotherapy-induced neutropenia and to evaluate the non-inferiority of the test drug relative to the originator.

**METHODS:**

This phase III non-inferiority study had a randomized, multicenter, and open-label design. The patients were randomized at a ratio of 1:1 with a follow-up period of 6 weeks for each patient. In both study arms, filgrastim was administered subcutaneously at a daily dose of 5 mg/kg body weight. The primary endpoint was the rate of grade 4 neutropenia in the first treatment cycle. The secondary endpoints were the duration of grade 4 neutropenia, the generation of anti-filgrastim antibodies, and the rates of adverse events, laboratory abnormalities, febrile neutropenia, and neutropenia of any grade.

**RESULTS:**

The primary efficacy analysis demonstrated the non-inferiority of the test drug compared with the originator drug; the upper limit of the 90% confidence interval (CI) for the rate of neutropenia between the two groups (12.61%) was lower than the established margin of non-inferiority. The two treatments were similar with respect to the secondary endpoints and safety.

**CONCLUSION:**

The efficacy and safety profile of the test drug were similar to those of the originator product based on the rate of grade 4 neutropenia in the first treatment cycle. This study supports Anvisa's approval of the first biosimilar drug manufactured by the Brazilian industry (Fiprima®).

## INTRODUCTION

Neutropenia is one of the most common chemotherapy-related adverse events, with a mortality rate of 10%, and it increases the costs associated with cancer treatment 1,2. Moreover, neutropenia is a severe limiting factor during chemotherapy 3 and may lead to decreased dosages and reduced treatment effectiveness 4,5, especially when chemotherapy is indicated for curative purposes, as is the case with the adjuvant treatment of breast cancer 6.

There is a direct correlation between absolute neutrophil count (ANC) and the risk of opportunistic infections in cancer patients undergoing chemotherapy 3. Counts below 500 neutrophils/mm^3^ are associated with an increased risk of infection or fever without the objective identification of microorganisms. It is estimated that 25%–40% of patients without previous treatment develop febrile neutropenia 7, which is defined as an ANC <500/mm^3^ and an oral temperature ≥38.2°C 8. This is categorized as grade 4 neutropenia on the 0–5 scale that is currently used for this adverse event 9.

The risk of febrile neutropenia varies according to the specific chemotherapy regimen 10. In patients with febrile neutropenia, toxicity commonly occurs during the initial chemotherapy cycles 11, reaching a maximum intensity between 7 and 14 days after initiating chemotherapy. This risk depends on numerous other factors, including tumor type and patient characteristics, such as age and performance status 1,5,12.

The use of broad-spectrum antibiotics and hematopoietic growth factors is currently considered effective against chemotherapy-induced toxicity 8,10,13,14.

Granulocyte colony-stimulating factor (G-CSF) is the most commonly used hematopoietic growth factor for the prophylaxis of febrile neutropenia. The G-CSF class of therapeutic agents was developed by isolating, purifying, and cloning this hematopoietic regulatory factor 15. The human G-CSF gene was inserted into *Escherichia coli* using recombinant DNA technology to produce Filgrastim, which has been approved for clinical use since 1991 16. Because G-CSF receptors are expressed solely in precursor and mature myeloid cells, the mechanism of action of filgrastim is selective; it stimulates the proliferation, differentiation, and activation of the neutrophil lineage, as well as shortens the neutrophil maturation period.

Recently, a pegylated formulation of filgrastim was released that reduced the renal clearance of the drug and increased the terminal half-life compared with unpegylated filgrastim 17. Pegylated filgrastim is administered in a single dose per chemotherapy cycle, and its effectiveness in shortening the duration of neutropenia is similar to that of daily filgrastim 18,19. Therefore, both forms of filgrastim can be used for the prophylaxis of febrile neutropenia.

Currently, the American Society of Clinical Oncology, the European Organization for Research and Treatment of Cancer, and the National Comprehensive Cancer Network 14 recommend primary prophylaxis with G-CSF when the expected rate of febrile neutropenia is ≥20%, regardless of the impact of other factors 10,20. This expected rate of febrile neutropenia at which primary prophylaxis with G-CSF is recommended was recently reduced from the previous rate of 40%.

Primary prophylaxis for febrile neutropenia can also be given to patients at high risk of neutropenic complications, even when the expected rate of febrile neutropenia is <20%. The high-risk factors include age >65 years, low performance status, cytopenia due to tumor-induced impairment of the bone marrow, active infections, or open wounds. Secondary prophylaxis is indicated for patients who previously experienced febrile neutropenia and for those whom a lower chemotherapy dose or a prolonged interval between chemotherapy cycles may be associated with worse overall or disease-free survival 10.

A previous study of experimental filgrastim produced by Eurofarma demonstrated comparability in terms of both *in vitro* biological activity and *in vivo* (rodent) toxicology and pharmacodynamics. A phase I, single-center, randomized trial was undertaken to demonstrate the equivalence of Eurofarma filgrastim and Roche filgrastim in terms of pharmacokinetic characteristics. Eurofarma filgrastim was well tolerated with no additional safety concerns compared with Roche filgrastim. Eurofarma filgrastim is bioequivalent to Roche filgrastim with regard to pharmacokinetics 21.

The aim of this study was to compare the efficacy and safety of two filgrastim formulations for controlling chemotherapy-induced neutropenia and to evaluate the non-inferiority of the test drug in relation to the originator.

## METHODS

This phase III, non-inferiority, randomized, open-label, multicenter study compared two products containing filgrastim: the test drug (Fiprima®, Eurofarma) and the originator drug (Granulokine®, Roche). The primary endpoint was the rate of grade 4 neutropenia in the first treatment cycle (considering the lower ANC value). The secondary endpoints included the rates of febrile neutropenia and neutropenia of any grade, the duration of grade 4 neutropenia, the generation of anti-filgrastim antibodies, and the frequency of adverse events and laboratory abnormalities.

This study was approved by the Research Ethics Committee of the participating institutions. All the participants provided written informed consent before undergoing any procedure in accordance with the Brazilian standards of Good Clinical Practice and the Declaration of Helsinki.

The selected patients included those with breast cancer stage II to IV; those with an indication for full-dose chemotherapy with one of the two eligible regimens (TAC regimen: 75 mg/m^2^ docetaxel, 50 mg/m^2^ doxorubicin, and 500 mg/m^2^ cyclophosphamide; or AT regimen: 75 mg/m^2^ docetaxel and 60 mg/m^2^ doxorubicin); those with no more than one previous chemotherapy regimen for metastatic disease with a performance status of 0 or 1 on the Zubrod scale and adequate organ function; and those aged ≥18 years who signed the informed consent form.

The exclusion criteria were as follows: an expected requirement for prophylactic or therapeutic antibiotics, antifungals, or antivirals in the first chemotherapy cycle; previous radiotherapy involving the pelvis or radiation in any body region in the 6 weeks prior to randomization; a history of receiving a bone marrow transplant; other tumors or serious comorbidities; recent participation (<12 months) in other clinical studies involving medication of any type or in interventional studies; an intolerance or allergy to any component of the filgrastim formulations evaluated in this study; and pregnant or lactating women.

The main demographic characteristics of the patients in the per-protocol (PP) and intention-to-treat (ITT) populations are shown in Table 1 according to treatment arm.

After stratification according to clinical site and the previous use of chemotherapy, the patients were randomized at a ratio of 1:1 to receive filgrastim (Fiprima® or Granulokine®); the follow-up period for each patient was 6 weeks, except in cases of death, withdrawal of consent, or loss to follow-up. In both study arms, filgrastim was administered subcutaneously at a daily dose of 5 mg/kg body weight until the ANC recovered to ​​≥10,000/mm^3^ or until the 15^th^ day of the chemotherapy cycle (V14), whichever occurred first.

The patients were evaluated at the screening visit (SV), at the randomization visit (RV, 7±3 days after the SV and 3±3 days before the first chemotherapy dose), and at daily visits during treatment. Filgrastim was administered on the odd visits (V1 to V13, such that V1 occurred on day 2 of the chemotherapy cycle), whereas on even visits (V2 to V14), drug administration was accompanied by health and safety assessments and laboratory tests. The final visit (FV) occurred 3 weeks (±5 days) after the first day of chemotherapy.

### Statistical analysis

To assess the non-inferiority of the test drug compared with the originator drug, the 90% CI for the difference in the rates of grade 4 neutropenia between the two groups was initially calculated. Non-inferiority was defined as an absolute value of the upper limit of the CI <15%. In an exploratory manner, the 95% CI for the difference in the rate of grade 4 neutropenia between the two groups was also calculated.

Continuous variables are presented as the variation (minimum and maximum values), mean, standard deviation (SD), median, and interquartile range (25^th^ percentile [Q1] – 75^th^ percentile [Q3]). Categorical variables are described as absolute and relative frequencies. The Kolmogorov-Smirnov test with the Lilliefors correction was used to evaluate the standard distribution of the outcome variables. The Lilliefors correction was also used to adjust the estimated population parameters (mean and variance or SD).

Continuous variables with a normal distribution were compared using t-tests, whereas variables with a non-normal distribution were compared using the nonparametric Mann-Whitney test. Categorical variables were compared using the chi-square test and the test for equality of proportions with the continuity correction. In general, two-tailed significance levels of 5% indicated significant differences between the groups.

### Sample size calculation

To calculate the sample size for the proposed non-inferiority design, the historical incidence of grade 4 neutropenia after the first chemotherapy cycle was considered. This value was between 73% and 83% in studies involving the two eligible chemotherapy regimens. Considering a one-tailed alpha of 5% and a statistical power of 80% for the study to obtain a maximum absolute difference of 15% in the rate of grade 4 neutropenia between the two groups, and assuming that this rate would be 80% in the control group, 88 patients should be included in each study group. Assuming a dropout rate of approximately 20%, 110 patients were anticipated in each arm, for a total of 220 patients.

### Study population

**Figure 1:**
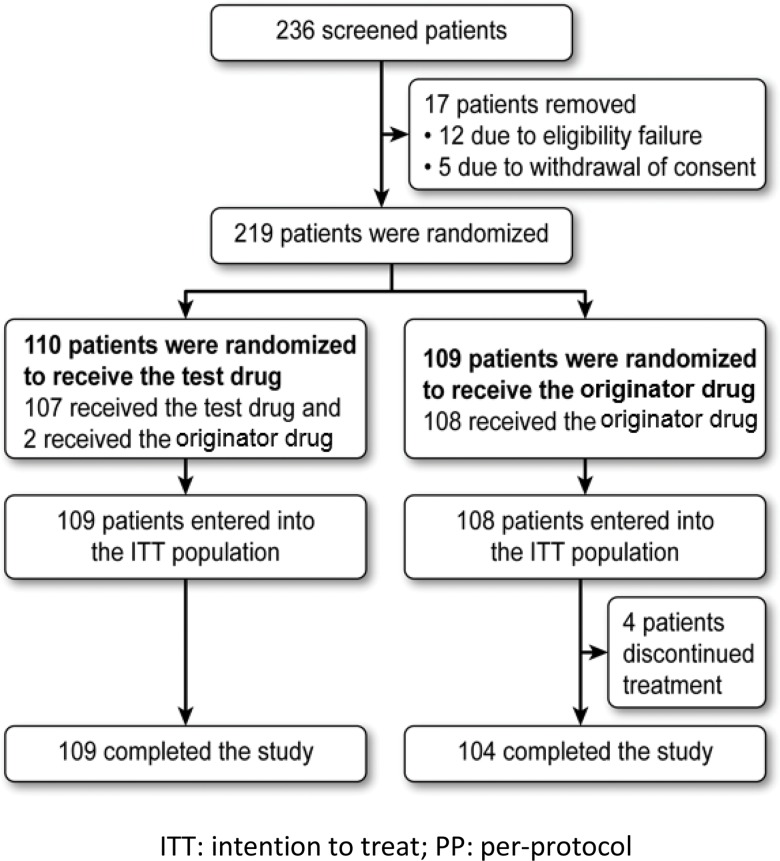
Flowchart of study population (CONSORT Diagram).

Between April 2011 and June 2012, 236 patients were screened, of which 219 were randomized. The reasons for randomization failure were withdrawal of consent (n=5) and failure to meet the eligibility criteria (n=12); these failures were due to inadequate organ function (n=8), the presence of serious comorbidities (n=1), radiation within 6 weeks prior to randomization (n=1), lack of an indication for chemotherapy with one of the eligible regimens (n=1), and performance status other than 0 or 1 on the Zubrod scale (n=1). Two randomized female patients did not receive at least one dose of the evaluated drugs; both patients discontinued the study prematurely, one for loss to follow-up and the other for withdrawal of consent. Of the 217 randomized patients who received at least one dose of the study treatment, two patients who had been randomized to receive the test drug instead received the originator drug. Figure 1 shows the flowchart of the study population according to treatment group and the total number of patients in each group.

Of the 217 eligible patients who received treatment, all belonged to the ITT and safety populations. Of these patients, 47 did not enter the PP population because they fulfilled at least one of the following criteria: received commercial filgrastim during the study period, were under medical surveillance, and did not perform all the tests specified in the study schedule (n=4); did not undergo an ANC evaluation at the SV (n=1); did not have at least 3 ANC results between V2 and V14 (necessary for evaluating the primary endpoint, n=1); or irregular administration of filgrastim (administration was discontinued before the ANC reached ≥10,000/mm^3^ after reaching the nadir; n=41). Therefore, the ITT, PP, and safety populations consisted of 217, 170 and 217 patients, respectively.

Four patients who received treatment, all from the originator drug group, prematurely discontinued the study due to non-adherence to the protocol or treatment (n=1), withdrawal of informed consent (n=1), use of medications not allowed during the study period (n=1), and death (n=1).

## RESULTS

The primary efficacy endpoint was determined by evaluating the non-inferiority of the test drug compared with the originator drug with respect to the rate of grade 4 neutropenia in the PP population. This rate was defined as the ratio of the number of patients with grade 4 neutropenia to the total number of patients in each group during the first chemotherapy cycle. Table 2 shows the percentage of patients with grade 4 neutropenia in the first chemotherapy cycle (between V2 and V14) stratified by treatment (test *vs*. originator) and the comparison of the rates of grade 4 neutropenia between the two treatment arms. There results was no significant difference (*p*=0.9971) in the rate of grade 4 neutropenia between the two groups in the first chemotherapy cycle.

**Figure 2:**
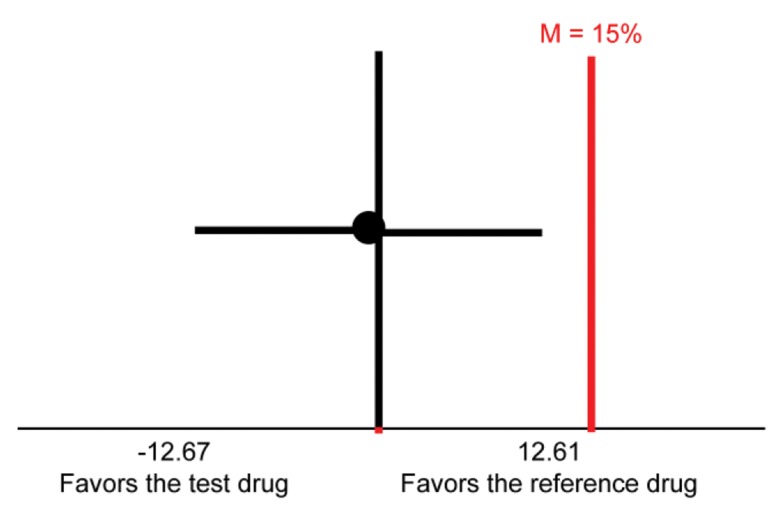
Primary analysis of efficacy in the PP population (test drug *versus* originator drug; n=170).

The 90% CI for the difference in the rate of grade 4 neutropenia between the test and originator drugs in the first chemotherapy cycle was between –12.67 and 12.61. The CI included zero, which indicates the absence of a significant difference in this rate between the two groups. To demonstrate the non-inferiority of the test drug compared with the originator drug, the upper limit of the 90% CI for the difference in the rate of neutropenia in the first cycle should be smaller than the margin of non-inferiority (M), which was set at an absolute value of 15%. Figure 2 presents the results in graphical form; the upper limit of the CI (12.61%) was below the M of 15% that was set during the sample size calculation. This analysis indicated the non-inferiority of the test drug in relation to the originator drug.

In addition to the 90% CI, the 95% CI for the difference in the average rate of grade 4 neutropenia in the first cycle was also calculated in an exploratory manner. This interval varied between –15.06 and 15.00 and also included zero, demonstrating the lack of a significant difference between the two groups.

With regard to the secondary endpoint, the number of patients with grade 4 neutropenia in the two groups in the ITT population was 56 (51.4%) for the test drug and 59 (54.6%) for the originator drug. The comparison of these rates indicated no significant difference between the two groups (*p*=0.6311), although the rate was slightly lower for the test drug than for the originator drug.

The rate of febrile neutropenia was defined as the ratio of the number of patients with febrile neutropenia to the total number of patients in each study group; only a single episode of febrile neutropenia during the study period was considered for each patient. The analysis of the PP population indicated rates of 3.49% and 2.38% in the test and originator drug groups, respectively; these rates were not significantly different (*p*=0.669). In the ITT population, the rates of febrile neutropenia were 3.7% and 2.8% for the test and originator drugs, respectively; again, these rates were not significantly different (*p*=0.710).

The rate of neutropenia of any grade was defined as the ratio of the number of patients with neutropenia of any grade (1 to 5) to the total number of patients in each group; a single episode of neutropenia at its worst grade in the evaluated cycle was considered for each patient. The number of patients with neutropenia in the ITT population was 99 (90.8%) for the test drug and 93 (86.1%) for the originator drug; there was no significant difference between the treatment arms regarding this variable (*p*=0.2768).

Table 3 lists the results for the grade of neutropenia, considering the worst grade for each patient in the ITT population between visits V2 and V14. The comparison between treatment groups using the chi-square test indicated no significant difference.

The duration of grade 4 neutropenia in the two treatment groups was estimated as the difference in days between the date of the onset of grade 4 neutropenia and the response “Yes” to the question regarding changes in the grade of neutropenia during the even visits. Because the evaluation was not conducted daily, the duration categories considered for this analysis were “within 2 days” and “3 to 4 days”. In the PP population, the number of patients with grade 4 neutropenia that lasted <2 days and 2–4 days was 39 (88.6%) and 5 (11.4%), respectively, in the test drug group and 40 (95.2%) and 2 (4.8%), respectively, in the originator drug group. In the ITT population, these numbers were 41 (89.1%) and 5 (10.9%) in the test drug group and 39 (92.9%) and 3 [7.15] in the originator drug group. The chi-square test indicated no significant differences in the duration of neutropenia between the two treatments in the two populations (PP, *p*=0.2631; ITT, *p*=0.5436).

**Figure 3:**
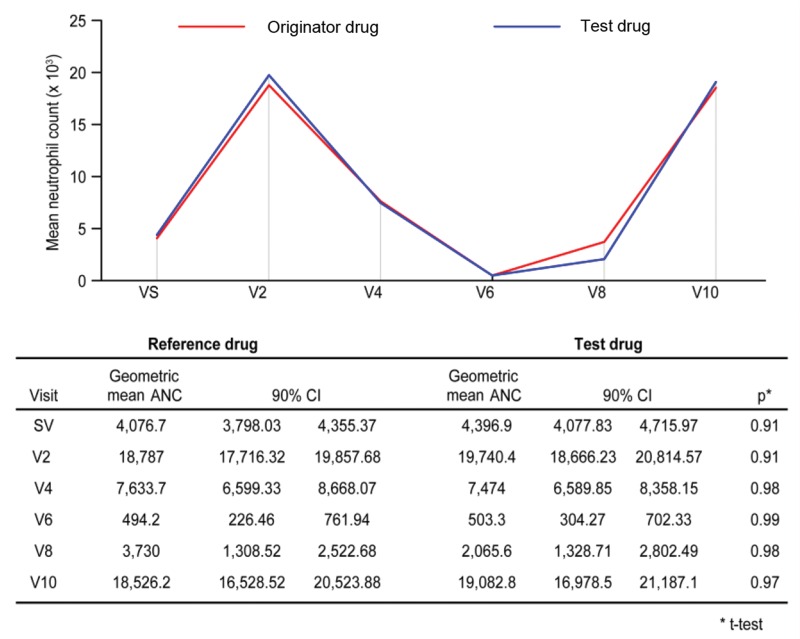
Geometric mean neutrophil count at each visit, with 90% CIs and *p*-values identified at each study visit.

In addition, the neutrophil count was assessed at each visit between the first and tenth days of study participation, and the data indicated that this value changed every other day. For each visit, a neutrophil recovery curve was generated considering the difference between the geometric mean neutrophil count in each group (Figure 3). The vast majority of patients received at least 9 daily filgrastim injections. The recovery curves fully overlapped.

The generation of anti-filgrastim antibodies was assessed by calculating the percentage of positive results (in the confirmatory test) for IgA, IgG, and IgM at the SV and V8 in the safety population. In the characterization phase, the samples with a positive result in the screening stage were analyzed; in the confirmation phase, the samples positive for any Ig in the characterization phase were analyzed. Patients with samples from a single visit were not included. The results indicated no significant difference in antibody production between the treatment groups.

### Safety results

In the safety population, 206 patients had mild-to-moderate adverse events during the study period: 101 in the test drug arm and 105 in the originator drug arm. The cases of neutropenia were not considered adverse events because neutropenia was a study endpoint and was therefore analyzed separately in the efficacy analyses.

The most common adverse events in the two groups were nausea (n=133; 61.3%), diarrhea (n=89; 41.0%), leukopenia (n=50; 23.0%), vomiting (n=49; 22.6%), and asthenia (n=48; 22.1%). For events that occurred in both groups, the relative difference between groups in the number of patients with at least one occurrence of that event was evaluated. Hypothesis tests for equality of proportions were conducted only for events with a relative difference of ≥10% between the groups. There were no significant differences in the frequencies of these adverse events between the groups.

Among the reported adverse events, 14 were considered strongly correlated with the study drug (11 in the test drug group and 14 in the originator drug group), 39 were considered possibly correlated with the study drug (26 in the test drug group and 13 in the originator drug group), and 15 were considered most likely correlated with the study medication (8 in the test drug group and 7 in the originator drug group).

In the safety population (n=217), nine patients had serious adverse events: three in the test drug arm (three records) and six in the originator drug arm (nine records). All the reported serious adverse events were considered clearly uncorrelated with the study drug.

There was one reported death during the study. In the database, this patient had documented abdominal pain and vomiting as serious adverse events; however, these events were considered uncorrelated with the study drugs.

## DISCUSSION

This study compared two filgrastim formulations to evaluate the non-inferiority of a test drug relative to an originator drug in the prevention and control of neutropenia associated with the TAC and AT chemotherapy regimens. The primary efficacy analysis of the primary endpoint in the PP population demonstrated the non-inferiority of the test drug compared with the originator drug; the upper limit of the 90% CI for the difference between groups in the rate of grade 4 neutropenia in the first treatment cycle (test drug–originator drug=12.61%) was lower than the absolute value of the pre-established M (15%).

Notably, the incidence of grade 4 neutropenia in both groups was lower than that used in the sample size calculation, most likely because blood counts were analyzed every two days. Consequently, an exploratory analysis was conducted using the 95% CI, and the result of this analysis corroborated the primary analysis. The latter analysis was broader and therefore more conservative, and its upper limit coincided with the adopted margin. Although these results are considered borderline with respect to demonstrating non-inferiority, the fact that the 95% CI did not exceed the M is further evidence in favor of the test drug.

It was not possible to demonstrate the superiority of the test drug because no significant differences were observed between the two formulations when the rates of grade 4 neutropenia in the first cycle were compared using the test for equality of proportions with the continuity correction (*p*=0.9971). The other comparative analyses revealed no significant differences between the two formulations in the other efficacy variables or in the toxicity profile.

In general, for the efficacy variables, the two drugs performed similarly with respect to the primary endpoint (rate of grade 4 neutropenia in the first treatment cycle) and to the secondary endpoints (rate of febrile neutropenia and duration of grade 4 neutropenia) evaluated in the PP and ITT populations.

Randomized trials have demonstrated the benefit of prophylactic filgrastim for the treatment of cytotoxic chemotherapy-induced neutropenia based on reductions in the following criteria: rate of febrile neutropenia; duration of grade 4 neutropenia; depth of the neutrophil nadir; number of hospitalizations; and rate of antibiotic use. Chemotherapy cycles of doxorubicin followed by docetaxel are known to be associated with a high incidence of hematological toxicity (rate of febrile neutropenia: 33%) 22.

Despite a similar patient cohort in terms of median age and treatment regimens, the rate of febrile neutropenia among the patients in our population was almost half that reported in pivotal studies. The reasons for this observation are not evident, but there are numerous plausible explanations, including fundamental differences in the patient population, supportive care, and other unmeasured factors. In our study, the women were chemotherapy naïve, with less vulnerable bone marrow, and more responsive than chemotherapy-pretreated patients. In contrast, 20% of the patients in previously published studies had received prior chemotherapy, a known risk factor for severe neutropenia. Nevertheless, there was no significant difference between the groups in our study 23,24.

With regard to safety, there were certain numerical differences between the groups in some of the analyses. However, there was no apparent pattern that indicated which treatment was less toxic. During the study period, there was one recorded death in the originator drug group. There was no significant difference in the frequency of adverse events between the two treatment groups. In addition, although no hypothesis tests were performed, our descriptive analyses suggested that the groups were similar with respect to changes in the measured laboratory parameters.

The efficacy of the test drug was similar to that of the originator drug with respect to non-inferiority in the rate of grade 4 neutropenia during the first chemotherapy cycle. There were no significant differences in the other efficacy and safety endpoints between the groups.

This study supports Anvisa's approval of the first biosimilar drug manufactured by the Brazilian industry (Fiprima®).

## FINANCIAL SUPPORT DISCLOSURE

This study was sponsored by Eurofarma Laboratórios S.A.

## OFF-LABEL INVESTIGATIONAL STUDY DISCLOSURE

This study is not an off-label investigational study.

## CONFLICT OF INTEREST

The authors report that this study received financial support from Eurofarma Laboratórios S.A. to analyze an investigational drug.

## AUTHOR CONTRIBUTIONS

Hegg R and Mattar A wrote and revised the manuscript. Hegg R, Mattar A, van Eyll Rocha S, de Matos Neto JN, Pedrini JL, Aleixo SB, Rocha RO and Cramer-Junior RP were responsible for the clinical trial and revising the manuscript.

## Figures and Tables

**Table 1:** Demographic characteristics of the patients according to treatment arm in the ITT and PP populations.

	ITT population (N=217)		PP population (N=170)	
Characteristic	Test drug (N=109)	Originator drug (N=108)	Test drug (N=86)	Originator drug (N=84)
Age (years)				
Mean ± SD	51.36±9.85	49.04±11.24	50.19±9.94	49.73±11.49
Range	30.11–76.39	22.30–78.10	30.11–76.39	22.30–78.10
Median	52.17 (44.94–58.09)	48.86 (42.81–55.58)	50.71 (42.91–57.19)	48.86 (43.44–59.03)
Ethnicity				
Caucasian	67 (61.5)	65 (60.2)	46 (53.5)	45 (53.6)
Black	13 (11.9)	13 (12.0)	11 (12.8)	9 (10.7)
Asian	3 (2.7)	2 (1.9)	3 (3.5)	2 (2.4)
Mixed	26 (23.9)	28 (25.9)	26 (30.2)	28 (33.3)

ITT: intention to treat; PP: per-protocol; SD: standard deviation. Mixed ethnicity included patients who marked “others” on the CRF. Age was calculated as the difference between the date of birth and the screening visit (SV) date.

**Table 2:** Rate of grade 4 neutropenia in the two treatment groups in the PP population (N=170).

Grade 4 neutropenia	Test drug (N=86)	Originator drug (N=84)	p*
No	42 (48.84%)	41 (48.81%)	0.9971
Yes	44 (51.16%)	43 (51.19%)	

(*) Test for equality of proportions with the continuity correction to compare the rate of grade 4 neutropenia between the two treatment groups.

**Table 3:** Rate of neutropenia considering the worst grade for each patient per treatment cycle in the ITT population (N=217).

Grade of neutropenia	Test drug (N=109)	Originator drug (N=108)	p*
0	10 (9.2%)	15 (13.9%)	0.5886
1	2 (1.8%)	3 (2.8%)	
2	14 (12.8%)	12 (11.1%)	
3	27 (24.8%)	19 (17.6%)	
4	56 (51.44%)	59 (54.6%)	

(*) Chi-square test. For each patient, the worst grade of neutropenia observed during the study period was considered.

ITT: intention to treat; PP: per-protocol
